# Intraoperative Cerebral Glioma Characterization with Contrast Enhanced Ultrasound

**DOI:** 10.1155/2014/484261

**Published:** 2014-06-12

**Authors:** Francesco Prada, Luca Mattei, Massimiliano Del Bene, Luca Aiani, Marco Saini, Cecilia Casali, Assunta Filippini, Federico Giuseppe Legnani, Alessandro Perin, Andrea Saladino, Ignazio Gaspare Vetrano, Luigi Solbiati, Alberto Martegani, Francesco DiMeco

**Affiliations:** ^1^Department of Neurosurgery, Fondazione IRCCS Istituto Neurologico “C. Besta”, 20133 Milan, Italy; ^2^Università degli Studi di Milano, 20122 Milan, Italy; ^3^Department of Radiology, Ospedale Valduce, 22100 Como, Italy; ^4^Department of Radiology, Ospedale di Circolo, 21052 Busto Arsizio, Italy; ^5^Department of Neurosurgery, Johns Hopkins Medical School, Baltimore, MD 21218, USA

## Abstract

*Background*. Contrast enhanced ultrasound (CEUS) is a dynamic and continuous modality providing real-time view of vascularization and flow distribution patterns of different organs and tumors. Nevertheless its intraoperative use for brain tumors visualization has been performed few times, and a thorough characterization of cerebral glioma had never been performed before. *Aim*. To perform the first characterization of cerebral glioma using CEUS and to possibly achieve an intraoperative differentiation of different gliomas. *Methods*. We performed CEUS in an off-label setting in 69 patients undergoing surgery for cerebral glioma. An intraoperative qualitative analysis was performed comparing iCEUS with B-mode imaging. A postprocedural semiquantitative analysis was then performed for each case, according to EFSUMB criteria. Results were related to histopathology. *Results*. We observed different CE patterns: LGG show a mild, dotted CE with diffuse appearance and slower, delayed arterial and venous phase. HGG have a high CE with a more nodular, nonhomogeneous appearance and fast perfusion patterns. *Conclusion*. Our study characterizes for the first time human brain glioma with CEUS, providing further insight regarding these tumors' biology. CEUS is a fast, safe, dynamic, real-time, and economic tool that might be helpful during surgery in differentiating malignant and benign gliomas and refining surgical strategy.

## 1. Background

Cerebral gliomas, both HGG and LGG, are a daunting challenge. Complete tumor resection remains the best treatment option as long as it can be achieved without neurological sequelae. The role of imaging techniques in surgical resection of brain lesions is crucial in every step of surgery: they help planning surgical strategy, provide orientation during surgery, and indicate tumor boundaries and relationships with eloquent areas and vital structures, thus enhancing precision, accuracy, and safety for the patients while maximizing resection [1–4]. In recent years we are witnessing an increased use of ultrasounds (US) in neurosurgery, as their reliability as an intraoperative tool for tumor detection has been shown in multiple studies [5–9]. US obviates the need for high costs and specialized surgical instruments. However, although standard US B-mode imaging is excellent for tumor localization, little information is provided regarding microcirculation and perfusion dynamics, even when integrated with Doppler sonography [10–13].

The use of contrast agents in medical imaging is aimed at enhancing differences and characteristics of various organs, vessels, and cavities, making their visualization more simple and efficient, and US are routinely used in diagnostic radiology. Contrast enhanced ultrasound (CEUS) is nowadays an established technique for many organs, as it allows, among other things, better detecting neoplastic lesions [14]. Furthermore, for their ability to highlight microcirculation, contrast agents are used in oncology in order to quantify the flow characteristics through an organ or tumor which differ according to the type of lesion and the organ involved [15–19]. The main clinically recognized application is the characterization of focal liver lesions [20]: CEUS with low-transmit power insonation allows real-time assessment of contrast enhancement and vascularity of focal lesions during the different dynamic phases, after injection of an intravenous contrast agent. Contrast agents containing microbubbles have been found to give the highest contrast with ultrasound scanning [21, 22]. The microbubbles consist of air or inert gas encapsulated in a layer of protein or polymers. Microbubbles are typically 5 micrometers in diameter, a similar size to red blood cells, and can therefore be transported into the smallest capillaries and across the lungs, thus allowing the visualization of the arterial system after venous injection. The pharmacokinetics of the microbubbles is quite different from that of contrast agents used for CT and MRI which generally diffuse in the interstitial space [15, 16, 23]. Second generation US contrast agents are clinically safe and well tolerated [24].

Given these technical features, it seems worthwhile and a promising effort to test this method for brain gliomas characterization and for its role in maximizing surgical resection, as already carried out by the radiological community for other organs. In fact, CEUS could provide us with further insight into glioma biology: being a dynamic and continuous modality it offers a real-time direct view of the degree of vascularization, microcirculation, flow distribution patterns, and tissue resistances of the different type of gliomas, adding all these pieces of information to the anatomical ones obtained with standard B-mode imaging.

Nonetheless its use in cerebral surgery has only been attempted few times so far [25–27], and there are no guidelines provided on this regard.

In this paper the authors describe, for the first time, the different patterns of cerebral gliomas enhancement using the CEUS technique, as compared with the lesion characterization achieved by using preliminary baseline US.

## 2. Material and Methods

### 2.1. Study Design and Patient Population

We performed intraoperative CEUS in an off-label setting in patients with supratentorial cerebral gliomas (both HGG and LGG) confirmed on preoperative MRI, undergoing craniotomy for tumor removal.

We included patients with no cardiopathy (New York Heart Association, NYHA I-II) and a good general status (ASA I-III).

All patients underwent preoperative assessment consisting of a thorough neurological and general conditions evaluation.

All patients were fully informed regarding their treatment and procedure and a written informed consent was obtained. The principles of the Declaration of Helsinki and the European Federation of Societies for Ultrasound in Medicine and Biology (EFSUMB) recommendations on CEUS [18, 28] had been followed.

### 2.2. Equipment and Contrast Agent

We used a last generation ultrasound device (MyLab, Esaote, Italy) with a 3–11 MHz linear probe.

The US system is equipped with Virtual Navigator software (MedCom, Germany) that permits fusion imaging between preoperative MRI and real-time intraoperative ultrasound imaging, allowing for neuronavigation.

As a contrast agent we used sulphur-hexafluoride, a second-generation ultrasound contrast agent (SonoVue, Bracco, Italy).

CEUS scanning is performed using contrast-tuned imaging (CnTI) technology that allows for real-time angiosonography, using second generation ultrasound contrast agents. Contrast-tuned imaging permits a selective synchronization of the US system to the signal produced by the microbubbles after transmission of a single-frequency pulse at the sulfur hexafluoride resonance frequency. The standard US imaging had been improved by CPI (combined pulsed imaging), a sophisticated algorithm based on a mix of high and low frequencies that improves B-mode penetration and resolution.

### 2.3. Procedure and Data Analysis

We perform a preoperative MRI based surgical planning. The craniotomy is performed with neuronavigation using standard preoperative MRI and coupled US using Virtual Navigator (Esaote, Italy).

The ultrasound apparatus is brought in and the 3–11 MHz intraoperative linear US probe (LA 332, Esaote, Italy) is placed in a transparent plastic surgical sterile sheath (Civco, USA), provided with US specific transducing gel.

After bone flap removal the US navigated probe is placed on the dura mater for scanning and standard B-mode imaging is acquired. All lesions are initially evaluated with B-mode imaging: they are defined as highly, mildly hyper-, iso-, and hypoechoic compared to normal brain parenchyma. Other lesion characteristics taken into account are diffuse or circumscribed appearance, homogeneous versus heterogeneous lesions, and presence of cystic/necrotic areas. The lesion is then identified on the two axes and measured. The lesion is also localized with neuronavigation on the corresponding coupled MRI.

Intraoperative CEUS is performed with the linear probe using low-power insonation and the obtained harmonic signals transduced with CnTI algorithm that allows for real-time and continuous imaging. Before microbubble contrast agent injection the focus is positioned below the level of the lesion. The contrast agent (SonoVue, Bracco, Italy) is injected intravenously by the anesthesiologist, as a bolus of 2.4 mL (5 mg/mL), followed by a flush of 10 cc. saline. The timer is started after UCA injection and perfusion dynamics is described starting from UCA arrival in major vessels; digital cine clips are registered continuously during baseline US scanning and during the different vascular phases.

After UCA injection, a first intraoperative qualitative analysis was performed, aimed at determining whether a contrast enhancement was detectable for every lesion and at its afferent and efferent vessels visualization. Data were also stored in the US device for offline analysis.

An offline data analysis of the CEUS cine clips was performed using a semiquantitative assessment, following the EFSUMB guidelines. Gliomas patterns of contrast enhancement (CE) were evaluated following the EFSUMB guidelines: timing (arterial and venous phase (time is given as range)), degree of CE (low, mild, and high; comparison with brain parenchyma), and contrast distribution (centripetal/centrifugal pattern, visibility of afferent/efferent vessels, intralesional vessels, and cystic/necrotic areas).

All data obtained by online and offline analysis were correlated with histopathology.

## 3. Results

Our population consisted of 69 patients (mean age 49 years; age range 12–71 years) who underwent surgery for supratentorial cerebral glioma. Histopathological data showed 47 HGG and 22 LGG. We further divided the two groups in two other subgroups: HGG group was composed of 36 glioblastomas (GBM) and 11 anaplastic astrocytomas (ANA). LGG group had 18 astrocytomas (ASTRO) and 4 oligodendrogliomas (OLIGO). Ultrasound findings were correlated with histopathology.

On standard US B-mode imaging glioblastoma (*n* − 47) appeared all hyperechoic compared to brain parenchyma, with a heterogeneous appearance composed of multiple well-defined nodular areas and others with diffuse margins. Size ranged from 3 to 7 cm of maximal diameter. All but three lesions had cystic/necrotic areas. Anaplastic astrocytoma (*n* − 11) ranged from 4 to 7 cm in diameter. All three appeared hyperechoic with a diffuse, dense texture, with some areas more hyperechoic compared to the rest of the lesion. No cystic/necrotic areas were noted. The brain/tumor interface was not everywhere clearly visible.

In the LGG group all lesions (*n* − 22) appeared mildly hyperechoic compared to brain parenchyma. Size ranged from 3 to 9 cm in maximum diameter. All lesions had a homogenous texture with blurred margins at the brain/tumor interface except one oligoastrocytoma which had a discrete appearance with clear border. Microcysts were visible in 5 cases only.

**Figure 1:**
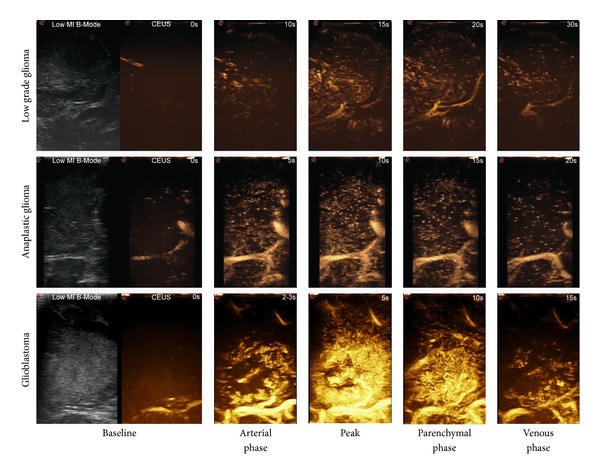
Time frame of how different grades of glioma are visualized with CEUS. In the first column of each row low mechanical index US and baseline CEUS (CA arrival – *t*_0_) are displayed; then different CEUS phases (time is displayed in the top right corner of each image) are displayed only. The image clearly shows the differences in terms of timing, degree of enhancement, and CEUS patterns for different types of glioma, with a continuous and dynamic modality.

**Figure 2:**
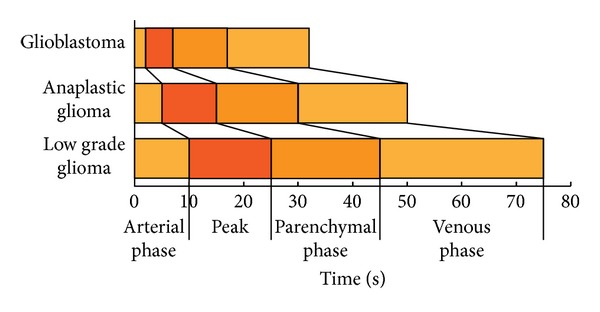
Schematic representation showing the differences in terms of timing and degree of enhancement (light orange: mild enhancement; dark orange: high enhancement) for different glioma grades.

After ultrasound contrast agent (UCA) injection different patterns were observed (Figures 1 and 2). All data are summarized in Table 1.

In the HGG group we further divided CE pattern into the two histological subgroups.

GBMs (*n* − 47) have rapid arterial and venous phase with a very fast arterial phase (2-3 seconds), chaotic transit of microbubbles within the lesion, and a CE peak at 5 seconds. CEUS transit time is very fast with a venous phase at 10 seconds. The major arterial supply was clearly visible, as well as the venous drainage system, almost invariably towards the periventricular zone. GBMs appear all hyperenhanced compared to normal brain parenchyma and have a very strong and intense contrast enhancement with a persistent parenchymal phase. They have an irregular and heterogeneous CE pattern with an alternation of nodular high contrast dense pattern with ring-like enhancement surrounding hypoperfused necrotic or nonperfused cystic areas. Many intralesional vessels are noted. We did not observe hypoperfused areas in only 6 cases, while 5 other cases only had small scattered hypoperfused areas. Tumor borders are better defined after CE than in standard B-mode imaging. GBMs showed a rapid refilling (around 3-4 seconds) after rapid sonication at high mechanical index sonication.

ANAs appeared to have a slower arterial phase compared to GBMs (10 sec), with a CE peak at around 15 seconds after UCA arrival. The transit of the microbubbles is slower and less chaotic and the venous phase is delayed as well (20–25 seconds), determining a lesion transit time of 5–10 seconds. Arterial supply and venous drainage are less identifiable than in GBMs. ANAs appear to have a diffuse hyperechoic pattern compared to brain parenchyma but have an initial mild and more homogeneous CE compared to GBMs, which is then reinforced during the parenchymal phase. We found a diffuse and persistent CE pattern in all cases, with scattered areas of higher CE in one case, the brain tumor interface. Few small hypoperfused areas were observed. Few intralesional vessels were observed. The border of the tumor is less identifiable than in GBMs. After high mechanical index sonication the replenishing kinetics is around 10 seconds.

In the LGG group, CEUS patterns for ASTRO were similar to ANAs. Nevertheless the vascular phases were slower, with an arterial phase at 15 seconds and a CE peak at around 20 seconds. The transit of the microbubbles appears even more steady (15–20 sec) with a venous phase after 30 seconds. Arterial supply is not always clear, as well as the venous drainage. ASTRO are mildly hyperechoic after UCA compared to brain parenchyma, and the tumor parenchymal phase is steady and uniform. Its CE pattern is dotted and homogeneous, with only two cases with microcystic areas. No intralesional vessels were noted. The replenishment kinetics is similar to the initial CE phase with a timing of around 10–15 seconds.

In OLIGOs we found similar features as in ANAs. In two cases the lesion was more well-defined, with faster arterial and venous phase, similar to GBMs, with an intralesion cyst.

## 4. Discussion

**Figure 3:**
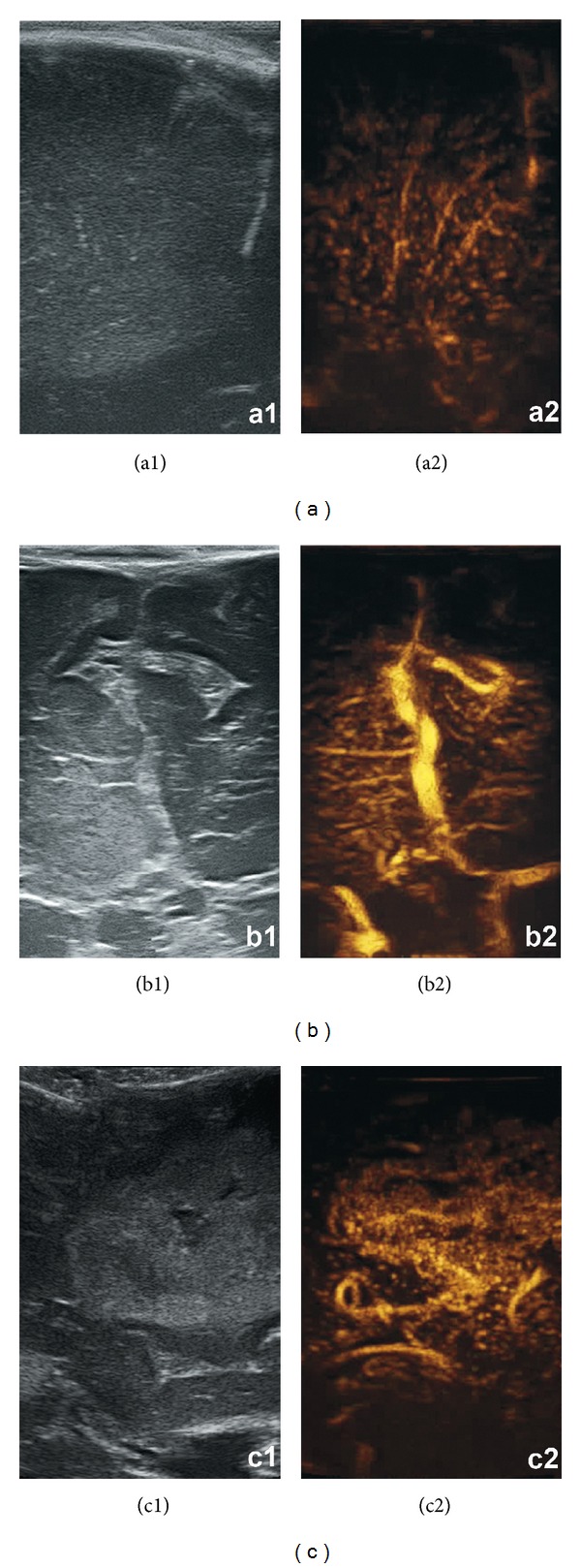
Comparison between standard gray-scale B-mode imaging and CEUS (resp., left and right picture in each panel) for different glioma grades (panel a: LGG, panel b: ANA, and panel c: GBM).

In this paper we performed the first intraoperative human cerebral gliomas characterization with the CEUS technique, as already had been performed for different lesions in other organs. The overall picture shows that in B-mode the main differences between lesions at different grades of malignancy are the degree of hyperechogenicity when compared to the surrounding parenchyma, the presence of cystic/necrotic areas, and a more or less defined brain/tumor interface. These findings account for the fact that the role of B-mode imaging is mainly limited in assisting tumor localization, providing only morphological information regarding the lesion, with little or no information about vascularization [7, 8]. Conversely, once enhanced, the tumor is highlighted and reveals other specific characteristics. These findings might possibly be related to their grade (Figure 3). For example, glioblastomas show rapid arterial and venous phase, a clearly visible arterial supply and venous drainage, and a very strong and intense contrast enhancement, with well-defined tumor borders. Lower grades were characterized by gradually less intense CE, less defined tumor borders, slower arterial and venous phases, poorly identifiable feeders and drainage, and a CE pattern progressively more homogeneous, accounting for the absence of necrotic/cystic areas and a minor amount of neoangiogenesis. Surprisingly, we observed a slighter but well-defined CE in low grades too, where preoperative MRI did not show any enhancement. We have been able to directly visualize each of the 69 lesions both in B-mode and after contrast infusion, regardless of its histology, thus making the comparison between the two modalities always possible. We also observed different morphologic and dynamic CEUS patterns, showing a very good correlation with histopathology (Figures 1 and 2). This confirms once more the reliability of this technique in assisting tumor resection. For the semiquantitative description of the lesions which followed the EFSUMB guidelines [18], consider parameters such as timing, degree of contrast enhancement (low, mild, and high) compared to normal brain parenchyma, diffuse or circumscribed appearance, homogeneous versus heterogeneous lesions, presence of cystic/necrotic areas, the pattern of CE, the timing of the different phases of CE, and microbubbles transit time within the tumor. Also, the arterial supply and the venous drainage were described when identified.

Of course CEUS cannot be considered as an alternative to histological examination, which remains the gold standard for diagnosis. Nevertheless, some of our cases considered on preoperative MRI as being low-grade gliomas were later histologically evaluated as anaplastic tumors. In these cases we intraoperatively observed some areas of focal CEUS enhancement. Therefore this technique can be helpful in guiding the surgeon through the choice of the areas for biopsy, thus possibly improving the accuracy of the final histological diagnosis.

**Figure 4:**
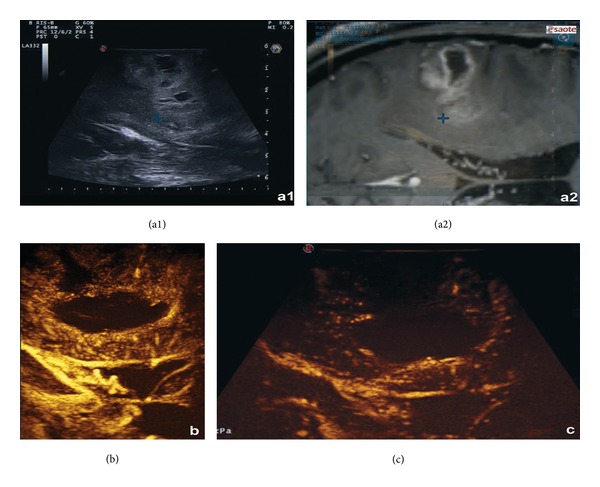
Intraoperative control of a right frontal GBM, using fusion imaging between intraoperative US (a1) and preoperative MRI (a2) linked via a navigated US probe with a virtual navigation system: (a1) shows a hyperechoic superficial lesion, with ill-defined borders and microcystic areas and in (a2) the corresponding MRI imaging is displayed. In panel (b) the B-mode imaging is enhanced with a contrast agent, showing a superficial nodular enhancement with a deeper ring enhancement delimitating a nonperfused necrotic central area. Medullary draining veins are also visible, draining towards the ependymal zone. In panel (c) the postresection control with CEUS shows the absence of the nodular ring enhancement, without contrast enhancement along the wall of the surgical cavity.

Another original aspect of our study is the unprecedented opportunity to conduct tumor resection under direct visualization and to highlight tumor boundaries and tumor remnants with CEUS during and after tumor resection. Performing CEUS prior to glioma resection will help differentiate tumor/edematous brain interface in HGG, and, as mentioned above, it will be able to show anaplastic areas within otherwise low-grade lesions (Figure 4).

After tumor removal in 9 GBMs we performed CEUS in order to highlight tumor remnants, thus possibly maximizing resection. In 3 cases we visualized CE areas which led to further tissue removal, whereas in the other 6 cases CE was not detected. Among the latter cases we also observed, in 2 cases, hyperechogenic areas in B-mode, suggestive for tumor residual which did not show any clear enhancement after contrast injection: in these cases the surgeon is facing one of the following possible situations. Either the area identified is a parenchymal contusion, which is hyperechoic due to the presence of blood cloth and this can be better discerned by visual inspection, or the hyperechoic area might represent a devascularized tumoral area: in fact, one should always keep in mind that microbubble contrast agents are confined to the intravascular compartment, unlike those used for CT or MRI enhancing, which mainly diffuse in the interstitial space. Therefore the closure of a tumor feeding artery leads to a noncontrasted area even when tumor is present. For the same reason, tumors with a greater degree of vascularization (i.e., GBMs) will be more clearly distinguishable from the surrounding healthy parenchyma, presenting with more defined borders as compared to less vascularized ones.

The capacity of CEUS to almost invariably visualize, in higher grades, feeding arteries and venous drainage is helpful for the intraoperative management of the surgical strategy, for example, by discerning whether a vessel is an actual tumor feeder, being as such safely subject to closure by cauterization, or is a vessel which is just passing through the tumor, heading to a portion of healthy parenchyma. Of course an early identification of a true feeder helps in controlling any major bleeding and in keeping the operatory field clean, while recognizing and preserving a vessel which is not strictly related to the tumor can prevent unexpected complications.

In the literature few studies using bedside transcranial US have been performed to evaluate the role of iCEUS in depicting cerebral tumors [29–31]. Harrer et al. used bedside transcranial CEUS prior to surgery and had been capable of discriminating brain lesions from the brain parenchyma and of partially describing them. Vicenzini et al. performed time intensity/curves using dedicated software: indeed time-intensity curves should be performed on a large sample size in order to provide statistically relevant results [28]. However tumor visualization was somehow poor due to transcranial US performed through a temporal window: despite being a well-established technique it suffers from limitations to tumor visualization due to the presence of the cranial vault in terms of both spatial resolution and tumor location. Intraoperative iCEUS permitting direct tumor visualization during surgery for brain tumor removal has been described only a few times [25–27]. Kanno and colleagues in 2005 evaluated that 40 brain tumors did not perform a continuous imaging because they used a first generation contrast agent. Engelhardt and colleagues in 2008 performed iCEUS during brain tumor removal, using a second generation contrast agent with specific algorithm on a very small and homogeneous cohort of patients (7 GBM patients). They also performed an offline analysis with time-intensity curves. He and colleagues evaluated 29 brain tumors using iCEUS but with some technical limitations: they used a phased array probe, with low frequency and a vast view-field, and the US imaging was performed in power Doppler modality instead of using a contrast specific algorithm, dramatically reducing both US spatial resolution and definition.

Of course, further studies are needed in order to assess CEUS role in tumor resection; the potentiality of this technique to maximize tumor resection has yet to be investigated and demonstrated. Comparison with other imaging modalities (such as MRI imaging T1 weighted GD) will be necessary for defining further advantages or limitations of this technique when compared with other imaging standards in actual practice. Moreover, further studies aimed at quantitative data analysis are mandatory for a rigorous validation of the method: these results will further improve CEUS characterization of cerebral gliomas and will also enhance knowledge of tumor biology, possibly leading to prediction of the responsiveness to therapy of a specific individual tumor, or to orientation during the choice of the best therapeutic option [32, 33].

In fact, one major limitation of this study is related to the semiquantitative nature of the analysis that has been performed on the data obtained by CEUS. Nondestructive US scanning, with specific algorithm performed with low acoustic power and sulfur hexafluoride, filled microbubble contrast agents, opens up to quantitative data analysis with dedicated software leading to real-time assessment and quantification of tumor contrast enhancement with microbubbles, measurement of organ transit time after microbubble injection, and analysis of tissue perfusion. Tissue perfusion may be quantified also by further evaluating the replenishment kinetics of the volume of microbubbles after their destruction in the imaged slice (using high mechanical index US), obtaining quantitative parameters related to local tissue perfusion [15, 19]. However, we believe that the first step, as already performed for other organs and in our previous study [34], is the qualitative and semiquantitative analysis; time-intensity curves providing quantitative data require very large cohorts of patients in order to achieve a statistical relevance, so we feel that a quantitative analysis would be of little value in this study [28, 29, 32, 33].

Finally, another limitation related to the technique might be that CEUS, as any method based on ultrasound imaging, is dependent on the experience of the examiner [35]. Moreover, in the neurosurgical community, few surgeons are accustomed to and specifically trained in the use of ultrasounds, and this is especially true for CEUS, since its use in neurosurgery is relatively new. Therefore, for a correct image interpretation a period of specific training is required.

## 5. Conclusions

In this paper we establish for the first time a CEUS characterization of cerebral gliomas.

By defining the paradigm of CEUS enhancement in gliomas, we add valuable biological information such as vascularization, microcirculation, and tissue perfusion dynamic and add these pieces of information to those obtained with standard B-mode imaging and might corroborate histological diagnosis.

Performing CEUS during glioma removal can be helpful for the surgeon to differentiate between tumor and edematous brain in HGG, while it will show anaplastic areas within otherwise considered low-grade lesions. After gross tumor removal CEUS might also be used in the future to highlight tumor remnants, thus maximizing resection avoiding neurological sequelae due to damaged healthy brain tissue. This may lead to reduction of hospitalization time and ameliorating prognosis, improving free survival rates and ameliorating the quality of life in glioma patients.

CEUS can be a fast, safe, dynamic, feasible and repeatable, relatively economic, precise, and accurate tool that helps in differentiating malignant and benign lesions and in maximizing tumor resection, thus improving free survival rates in glioma patients; we believe that CEUS is definitely a methodology to further understand and develop in glioma surgery and the expected results will certainly integrate scientific excellence possibly leading to better treatment for cerebral tumors bearing patients.

## Figures and Tables

**Table 1:** Summarizing CEUS features of different grades of human cerebral gliomas.

Brain Lesion	Number of pts.	Echogenicity	Appearance	Cystic areas and/or necrosis	Arterial phase	CEUS peak	Venous phase	CE
Low-grade glioma	22	Iso/hyperechoic	Diffuse; homogeneous	Small/microcysts	15′′	20′′	30′′	Mild
Anaplastic glioma	11	Iso/hyperechoic	Diffuse; homogeneous	Small/microcysts	10′′	15′′	20–25′′	Mild/high
Glioblastoma	36	Hyperechoic	Diffuse/circumscribed; heterogeneous	Large necrotic areas	2-3′′	5′′	10′′	High

## Abbreviations

CEUS: Contrast enhanced ultrasound
US: Ultrasound
CE: Contrast enhancement
MRI: Magnetic resonance imaging
WHO: World Health Organization
HGG: High grade glioma
LGG: Low grade glioma
GBM: Glioblastoma (WHO grade IV)
ANA: Anaplastic astrocytoma (WHO grade III)
ASTRO: Astrocytoma (WHO grade II)
OLIGO: Oligodendroglioma (WHO grade II)
UCA: Ultrasound contrast agent.
